# Experimental Investigation for Monitoring Corrosion Using Plastic Optical Fiber Sensors

**DOI:** 10.3390/s24030885

**Published:** 2024-01-29

**Authors:** Liang Hou, Shinichi Akutagawa, Yuki Tomoshige, Takashi Kimura

**Affiliations:** 1 Department of Civil Engineering, Kobe University, 1-1, Rokkodai-cho, Nada-ku, Kobe 657-8501, Japan; cadax@kobe-u.ac.jp; 2 Engineering Department, JFE Shoji Terre One Corporation, 2-1, Otemachi 2-Chome, Chiyoda-ku, Tokyo 100-0004, Japan; yuki.tomoshige@terre-1.co.jp (Y.T.); takashi.kimura@terre-1.co.jp (T.K.)

**Keywords:** corrosion detection, color change, plastic optical fiber sensor, graphic image analysis

## Abstract

The timely and cost-effective identification of the onset of corrosion and its progress would be critical for effectively maintaining structural integrity. Consequently, a series of fundamental experiments were conducted to capture the corrosion process on a steel plate using a new type of plastic optical fiber (POF) sensor. Electrolytic corrosion experiments were performed on a 5 mm thick steel plate immersed in an aqueous solution. The POF sensor installed on the upper side of the plate and directed downward detected the upward progression of the corrosion zone that formed on the underside of the plate. The results showed that the POF sensors could detect the onset of the upward-progressing corrosion front as it passed the 1 and 2 mm marks related to the thickness of the corroded zone. The POF sensors were designed to optically identify corrosion; therefore, the data obtained by these sensors could be processed using a newly developed graphic application software for smartphones and also identified by the naked eye. This method offered an easy and cost-effective solution for verifying the corrosion state of structural components.

## 1. Introduction

A precise understanding of the corrosion status of metals is crucial for accurately assessing the structural integrity of all construction employing metal components. As metal corrosion was an electrochemical process, it occurred at specific locations and times within an environment that was conducive to corrosion, leading to the discoloration of the affected area. If the corrosive environment persisted, corrosion would proceed across the surface and penetrate the metal. Depending on the extent of the corrosion, a structure linked to or in contact with a corroded region might undergo deformation. Research on the corrosion process and its monitoring has been conducted across numerous disciplines [1,2,3,4,5]. These methods included directly tracking the corrosive environment [6], analyzing electrochemical signals in metals that were generated or altered owing to corrosion [7,8,9,10,11,12,13,14,15], assessing specimen weight fluctuations induced by corrosion [16], evaluating structural deformation resulting from corrosion [17,18,19,20], and examining alterations in the wave propagation characteristics of metal components owing to corrosion [21,22]. While the aforementioned methods demonstrate their ability to accurately monitor corrosion in specific scenarios, reliable execution requires highly technical expertise for monitoring and associated equipment costs.

Some approaches also aim to visually detect alterations in the condition of metal surfaces after corrosion [23,24]. However, these methods were confined to the exterior of readily observable metals and were yet to advance to the point of acquiring data regarding the progression of corrosion within the metal.

As previously outlined, research and development related to the detection of corrosion in metals has been approached from multiple perspectives, resulting in the creation of highly precise techniques for acquiring specific data (such as the presence of corrosion and its location, extent, and influence on neighboring structures) from each of these perspectives. However, to efficiently manage the extensive infrastructural assets in the forthcoming era of big data, methods should extend beyond the existing expensive and high-precision techniques. Therefore, a strategic approach should involve the use of sufficient cost-efficient sensors while integrating them with additional data to obtain insights into corrosion phenomena, thereby enabling a comprehensive evaluation of the condition of the infrastructure in general [25].

We introduced a novel corrosion-detection sensor utilizing plastic optical fiber (POF) to address these challenges. This sensor is comprised of a POF cable for illuminating the detection site and another POF cable for capturing changes in the reflected light, mainly alterations in brightness, at that location. The reflected light, influenced by corrosion events, was analyzed through an image processing application installed on a smartphone to confirm the occurrence of corrosion. Moreover, we demonstrated that the light affected by corrosion could be visually verified. The proposed POF sensor was cost-effective and user-friendly, utilizing plastic materials for all installation components. We initially presented the fundamental concept of the POF sensor. Subsequently, through both basic and applied experiments, we established its capability to detect corrosion phenomena within a steel plate at designated observation points. Ultimately, we illustrated the potential of the sensor to monitor corrosion across numerous observation points at varying depths within a steel plate at a low cost.

## 2. Basic Functions of Plastic Optical Fiber (POF) Sensors and Methods of Optical Data Analysis 

### 2.1. Past Applications of POF Sensors in the Civil Engineering Field

POFs exhibit greater flexibility and larger diameters than do glass optical fibers. They are actively advocated for widespread adoption across diverse domains as an optical transmission medium, offering easy connectivity and handling for ordinary users [26,27,28]. Several researchers had compiled a set of new ideas on diverse POF sensors for monitoring applications in civil engineering [29]. Initially, the POF sensor was utilized by bonding two POFs together; one received light input, while the other captured the light reflected from the tip. This sensor proved to be effective at discerning stationary and moving soil particles, like sand, providing valuable insights into water penetration within dry sand [30] and the ground’s movement as an early indicator of slope failure [31]. Subsequently, another experiment involved bonding two POFs adjacent to each other and cutting their tips at a 45° angle so that their tip looks like the tip of a sharpened pencil. These sensors were used to observe alterations in the ratio of light emitted outside versus that reflected inside the POF on the oblique surface, which was found to be influenced by the refractive index of the surrounding material. This facilitated the determination of moisture-related information at the sensor’s tip, enabling the acquisition of data concerning groundwater inflow, freeze-thaw cycles in groundwater [32], and the progressive hydration reaction and water content reduction during concrete curing [33]. Despite the diverse applications of POF sensors in civil engineering, sensors specifically designed for effective metal corrosion detection have not been developed yet. 

### 2.2. Characteristics of the POF Utilized in Corrosion Detection

**Figure 1:**
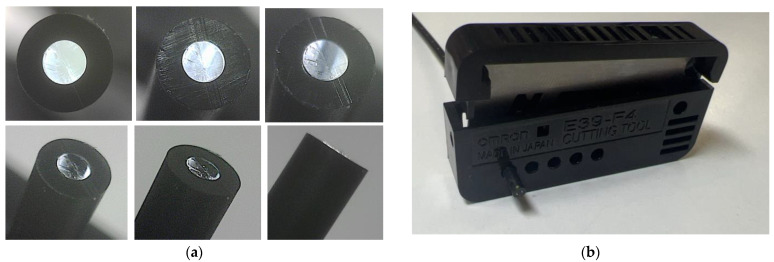
Cross section of a POF cable and a POF cutter: (**a**) Cross section of a POF cable taken from various angles; (**b**) A compact POF cutter (Model E39-F4 produced by OMRON). After inserting a POF cable into a hole, the blade can be pressed down to cut it.

Various types of POFs with different sizes and internal structures exist. The POF utilized in this study was a step-index polymer product with a consistent refractive index at the core. The model number of the POF is PGS-CD1001-22-E, and it is produced by TORAY. The core had a diameter of 0.98 mm (refractive index: 1.49) for transmitting light, whereas the surrounding cladding was 0.02 mm thick (refractive index: 1.41). The transmission loss was 150 dB/km (illuminated with a 650 nm wavelength light). The POF cable was encased in polyethylene with a total outer diameter of 2.2 mm, as shown in Figure 1. Creating a POF sensor involves cutting processes of POF cables to the prescribed length and then either introducing light into the end face or recording the light emitted from it. While it was feasible to polish the cut surface to achieve complete flatness, this incurred excessive time and cost, impeding the technology’s widespread societal adoption. In Figure 1a, surface photos taken from various angles of a POF cable are shown. To make this cut surface, the POF cutter shown in Figure 1b was used. These surfaces were mostly perpendicular to the POF axis, yet they retained microscopic irregularities. As these irregularities varied slightly between POFs, fabricating multiple sensors of the same type introduced some variance in measured values. Nonetheless, this divergence in sensor characteristics isn’t critical. The stability and variability of data from their respective initial values offered insight into the occurrence and progression of corrosion conditions. 

It is important to highlight that the objective of utilizing POF in measurements was to track alterations in the light captured by the POF from its initial value within specific conditions. Therefore, precise determination of the POF’s length would not be essential.

### 2.3. Angles of Light Entering and Exiting the POF

**Figure 2:**
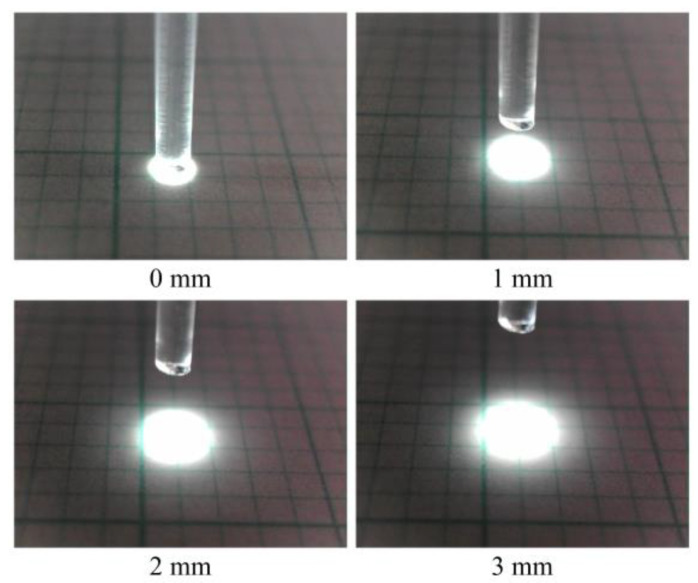
Size comparison of illuminated zones with respect to the vertical distance between the scale sheet and the tip of POF with a length of 1.5 m.

When light entered the POF, it underwent multiple reflections owing to the contrast in the refractive indices of the core and cladding. Consequently, the light emitted from the POF tip exhibited marginal dispersion. A POF tip that was positioned at a distance of 3 mm from the grid paper revealed a light spot with a diameter of approximately 2.5–3 mm, as shown in Figure 2.

**Figure 3:**
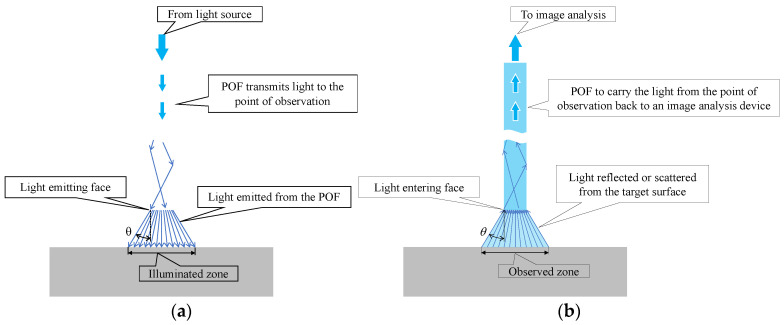
Identification of the illuminated and observed zones with respect to paths of light: (**a**) Paths of light exiting the POF; (**b**) Paths of light entering the POF.

Figure 3 illustrates the path of light. Light within the POF was propagated and emitted from the POF tip, spreading to a maximum angle of approximately 30° (with a marginal variation depending on the flatness of the end face), as shown in Figure 3a. It illuminated the surface of the object under examination. Conversely, when the light was reflected or scattered from the object’s surface and traveled in random directions, a part of reflected or scattered light followed the path shown in Figure 3b and entered the POF. This light reflected the color of the observed area, which spread with a maximum angle of 30° from the tip of the POF.

### 2.4. Fundamental Principle of Reading State Changes on the Surface of an Object

**Figure 4:**
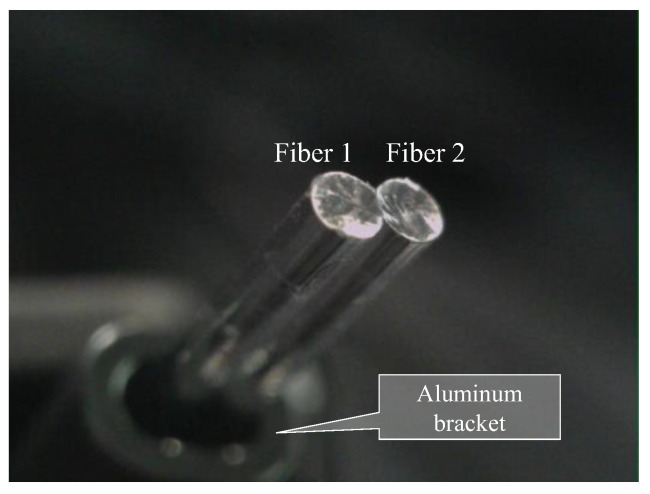
R2 sensor comprising two POF cables with a length of 1.5 m.

**Figure 5:**
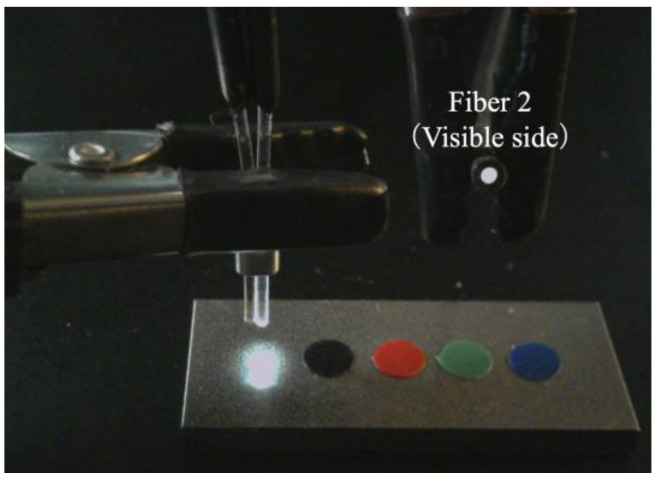
R2 sensor and a steel plate with vinyl tapes of different colors.

**Figure 6:**
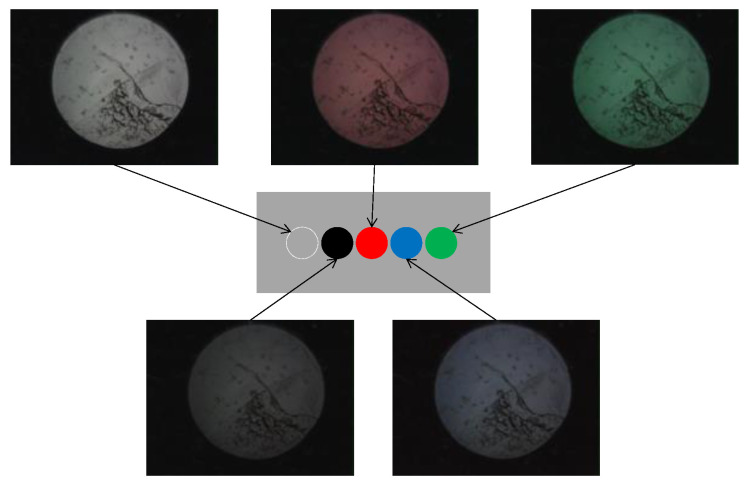
Images of light captured by Fiber 2.

Based on the observed light behavior at the POF tip, an introductory experiment employing two POF cables to show a basic and important ability to recognize change in the appearance or color of the observed spots prepared on the surface of the metal plate, was performed. Initially, two POF cables, namely Fibers 1 and 2, were prepared, and their tips were placed adjacent to each other after stripping approximately 20 mm of the insulation (as illustrated in Figure 4). This sensor, comprising two POF cables, was denoted as an R2 sensor. The name arose from its capacity to detect alterations in the condition of an arbitrary object based on the reflection of light from the surface in front of the sensor. As illustrated in Figure 5, circular vinyl tape segments (5 mm in diameter) in black, red, green, and blue colors were fixed to a steel plate (15 mm wide, 38 mm long, and 3 mm thick). Light reflected from the surface of the steel plate (without tape), positioned approximately 3 mm in front of the R2 sensor, was directed onto Fiber 2, establishing an initial state that could be examined from the opposite end of the POF. Subsequently, the steel plate was incrementally shifted to the left, and the reflected light was recorded by Fiber 2 at each stage as the R2 sensor tip passed above the tapes of distinct colors, as shown in Figure 6. The changes in the optical conditions in the area in front of the R2 sensor, primarily related to color changes, were recorded. This suggested that the R2 sensor can identify variations in the optical conditions attributed to metal corrosion.

### 2.5. Digital Processing of Light Captured by the POF

**Figure 7:**
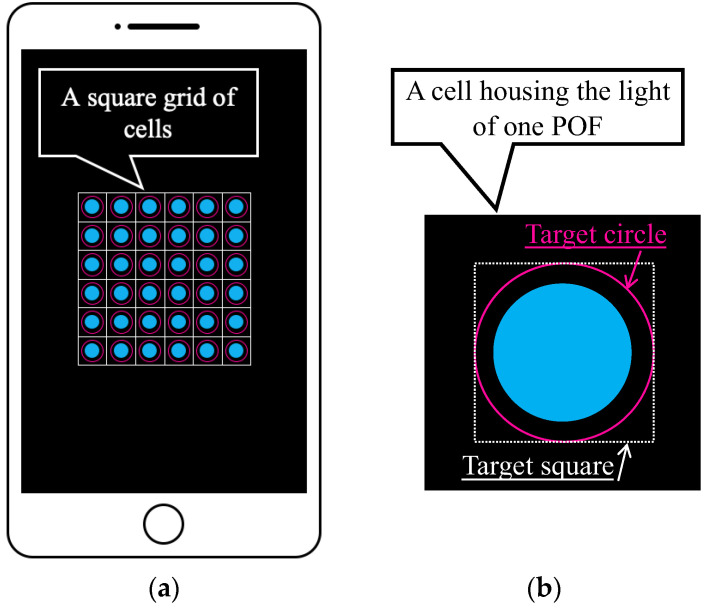
Basic features of the graphic application software: (**a**) Multiple POF sensors captured using a smartphone (an image) where each POF was housed in one of the cells in the square grid of cells; (**b**) Definition of the target square for which average values of *R*, *G*, and *B* were calculated.

The fundamental capability of the R2 sensor to detect color changes resulting from metal corrosion was visually confirmed. However, the light data collected by the POF sensor had to be processed digitally for more comprehensive tracking of the occurrence and progression of corrosion. Therefore, a new application software [34], hereinafter referred to as the “image processing application”, that utilized the image processing functions of a smartphone was developed. The image processing application could capture the light obtained through the POF and analyze the data using either the integrated camera of the smartphone or an external camera. This approach allowed precise monitoring suitable for engineering applications in various scenarios, including tasks such as color identification, displacement measurement, tilt measurement, and moisture detection on the ground [34]. The basic features of the system are illustrated in Figure 7.

As shown in Figure 7a, the light captured by multiple POF sensors is accurately distributed within individual cells in a square grid of cells. In this scenario, a target square inscribed within a user-defined target circle (indicated by the pink circle in Figure 7b) in each cell was first established as the square area representing one POF sensor. We assumed that the total number of pixels within this square is represented by n. Given that the fundamental data of the three primary light colors held by the *i*-th pixel are *R_i_* (red), *G_i_* (green), and *B_i_* (blue), the average values (*R*, *G*, and *B*) within the target square can be computed as follows:

(1) $$R=\frac{1}{n}\overset{n}{\underset{i=1}{\sum }}R_{i},$$

(2) $$G=\frac{1}{n}\overset{n}{\underset{i=1}{\sum }}G_{i},$$

(3) $$B=\frac{1}{n}\overset{n}{\underset{i=1}{\sum }}B_{i}.$$

Once these average values were acquired, the light intensity, denoted as *L*, can be defined as the index that characterizes the brightness of the light using the following formula:

(4) $$L=\sqrt{R^{2}+G^{2}+B^{2}.}$$

The image processing application employed in this study was designed for smartphones loaded with the Android operating system. Given that the *R*, *G*, and *B* values range from 0 to 255, *L* served as an index for defining the light intensity and fell within the range of 0 to approximately 441. The average values of the three primary light colors (*R*, *G*, and *B*) and *L* were influenced by the size of the measured circle. Note that these measures were not considered specific physical quantities of light captured by the POF sensor. Conversely, they served as indices that offer vital insights for tracking deviations from their initial values, enabling the detection and continuous monitoring of the progress of metal corrosion processes.

### 2.6. Appropriate Brightness of Light Used in the Proposed Scheme of Monitoring

**Figure 8:**
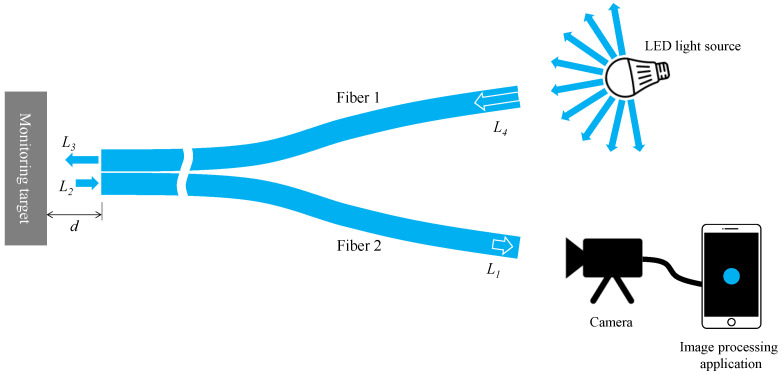
A typical layout of an experimental setup for corrosion monitoring by R2 sensor.

Considering the fundamental components explained earlier, the experimental setup’s basic layout for corrosion detection using the proposed R2 sensor is depicted in Figure 8. We first denoted *L*_4_ as the initial light intensity when a segment of emitted light from an arbitrary LED source entered Fiber 1. As this light traversed Fiber 1, reaching the observation point at its opposite end, the light intensity diminished to *L*_3_ (lesser than *L*_4_) due to inherent distance attenuation within the POF’s polymer material. Upon reflecting from the object’s surface and entering Fiber 2 as *L*_2_, a fraction of this light contained corrosion-related information. Subsequently, it traveled through Fiber 2, reaching its termination, labeled as *L*_1_ (lesser than *L*_2_) due to identical distance attenuation. This light, captured by a camera, underwent image data recognition via an image processing application on a smartphone. The final light intensity, denoted as *L*, was calculated following the method detailed in Section 2.5. Drawing from our experimentally-based expertise from the recent study [34], favorable outcomes typically arise when the smartphone records a final light intensity of approximately half or less than the maximum value (around 441). Consequently, during the corrosion detection experiment, adjustments encompassing the light source’s brightness, distance from the light source to Fiber 1, POF length, and distance from Fiber 2’s right end to the camera were made holistically. This ensured that the final measured light intensity *L* remained around half or less of its maximum value.

### 2.7. Optimal Distance between the R2 Sensor and the Measurement Object

**Figure 9:**
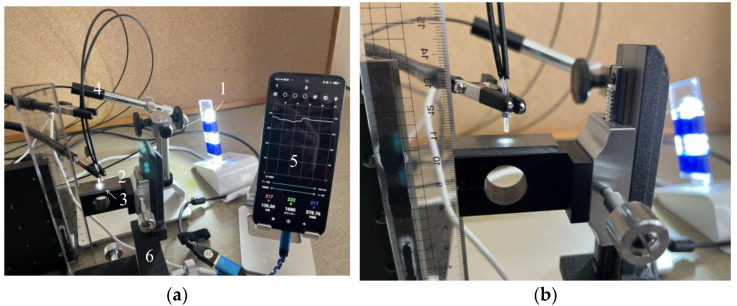
Experimental apparatus used to examine the optimal distance from the R2 sensor to a steel plate: (**a**) Experimental layout: (1) Light-emitting diode (LED) light. (2) R2 sensor. (3) Steel plate. (4) USB camera (AS ONE Corporation, USB Digital Microscope SDM200). (5) Smartphone with the graphic application software. (6) Three-dimensional stage; (**b**) Enhanced view of the R2 sensor and the steel plate.

The separation distance, denoted as d, between the R2 sensor and the object must be determined to allow proper observation of a target surface. Ensuring adequate illumination of the observed surface, particularly the area in front of Fiber 2, was crucial for achieving optimal light reflection into Fiber 2. If Fiber 1’s tip was positioned too close to the object’s surface, the illuminated area would be limited, resulting in reduced light entering Fiber 2. Conversely, placing Fiber 1 too far from the object’s surface will diminish both incident and reflected light. Consequently, we have chosen to experimentally determine the optimal distance for Fiber 2 to capture an adequate level of light. Figure 9 shows the experimental scheme used in this investigation, which was based on the concept shown in Figure 8. In this figure, the light emitted from the tip of the R2 sensor (2) was reflected by the surface of the steel plate (3) before entering Fiber 2. Subsequently, the light from the opposite end of Fiber 2 reached the image processing application (5) through a Universal Serial Bus (USB) camera (4). A three-dimensional stage (6) was used to lower the steel strip, while the separation distance, *d*, was altered from 0 to 6 mm.

**Figure 10:**
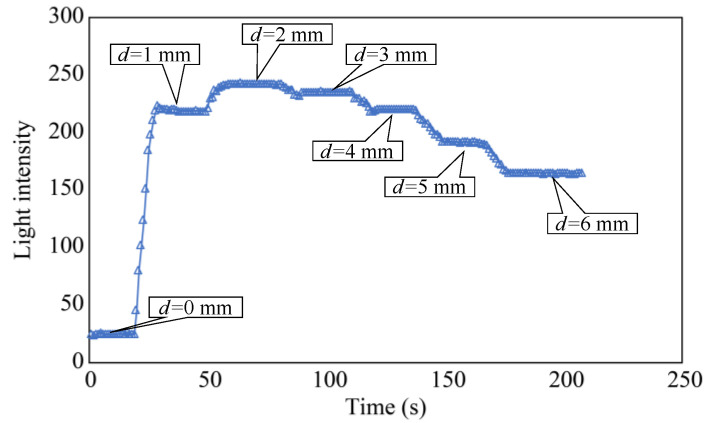
Light intensity recorded as the distance *d* was varied.

In the initial phase of the plate-lowering experiment, the separation distance, *d*, was set to zero and maintained for approximately 20 s. Subsequently, *d* was incrementally increased by 1 mm every 10 s and maintained for approximately 20 s. When *d* reached 6 mm, it was maintained at this value for approximately 30 s to complete the experiment. Figure 10 shows the light intensity *L* of the R2 sensor at this point, revealing that *L* was significantly higher within the range of *d* = 2–3 mm. Based on this trend, the standard separation distance, *d*, was determined to be 3 mm. This decision considered the convenience of the fixture used to create a more advanced sensor for subsequent experiments.

## 3. Method for Detecting the Depth of Corrosion

### 3.1. Comprehending the Occurrence and Progress of Corrosion

**Figure 11:**
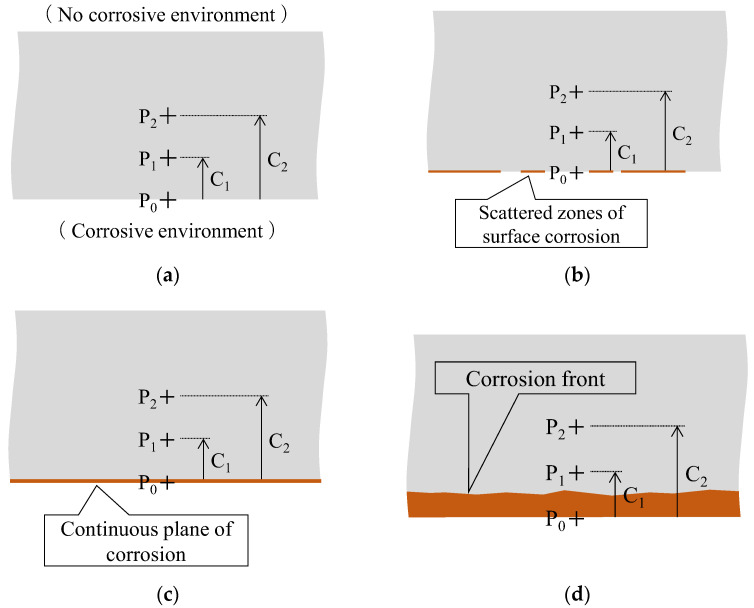
Scenario exhibiting the corrosion process in a steel plate: (**a**) Initial condition; (**b**) Scattered zones of corrosion on the lower surface; (**c**) Completion of corrosion across the lower surface; (**d**) Progress of the corrosion front in the upward direction.

The corrosion of metals manifested in diverse forms of development and propagation. However, we focused on the scenario illustrated in Figure 11. We considered a plate-like metal structure, and initially, the corrosive environment was presumed to be present only around the bottom surface and not the top surface (Figure 11a). Under these conditions, corrosion was first initiated randomly and at discontinuous areas on the bottom surface (Figure 11b); subsequently, it spread across the entire bottom surface (Figure 11c). The corrosion that extended across the entire bottom surface progressed in the depth direction over time, and the corrosion front gradually ascended (Figure 11d).

In this scenario, the corrosion monitoring strategy involved monitoring both the surface area affected by corrosion (as indicated in Figure 11b,c) and the progression of the corrosion depth within the metal plate (as represented by the expansion of the corrosion area from P_0_ to P_1_ at depth C_1_ and further to P_2_ at depth C_2_ in Figure 11d). Our primary focus was on the latter aspect. Therefore, we designed and conducted experiments with a sensor capable to detect the gradual growth in corrosion depth, for instance, from 1 to 2 mm.

### 3.2. Light Components Detected by the Sensor

Building on prior discussions, a sensor was designed to monitor the upward propagation of the corrosion phenomena beneath a steel plate. The design adhered to the following fundamental conditions:

- The sensor was placed in a downward direction in an installation hole with a diameter of 5 mm, located on the upper surface of the steel plate;
- A 3 mm separation existed between the sensor tip and the bottom of the installation hole;
- A transparent adhesive was used to fill the gap between the sensor tip and the bottom of the installation hole, as well as the space around the entire sensor and installation hole to prevent water infiltration.

In a practical sensor assembly process, employing distinct types of adhesives was essential: one for the sensor assembly (referred to as adhesive A) and another for securing the assembled sensor in the installation hole on the upper surface of the steel plate (referred to as adhesive B). Consequently, the R2 sensor, comprising two POFs, monitors the corrosion phenomena on the steel-plate surface through adhesives A and B.

**Figure 12:**
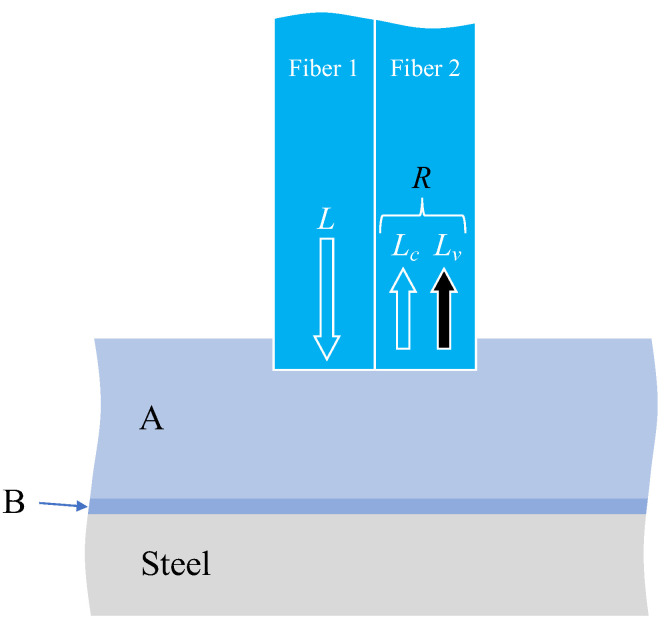
Various light paths associated with corrosion monitoring through two layers of adhesive materials.

The factors considered when attempting to observe corrosion on the surface of a steel plate through two adhesive layers, as illustrated in Figure 12, were explained subsequently. The light was reflected or scattered from the boundary surfaces between adhesive layers A and B, between adhesive B and the steel plate (steel), and from the actual surface of the steel material. Consequently, the observed light, *R*, in Fiber 2 was expressed as the sum of two components.

(5) $$R=L_{c}+L_{v}\;$$

Here, *L_c_* denotes the sum of the light scattered from the edge of Fiber 1, and the light reflected and scattered from the boundary between adhesives A and B, which is unrelated to the corrosion of the steel plate. In principle, this value remains constant. Conversely, *L_v_* signifies the light influenced by the corrosion of the steel plate and exhibits variability. Hence, when scrutinizing the light *R* captured by the image processing application, the state of steel corrosion must be assessed by considering the overall trend of *R* variation while acknowledging that some light encompasses aspects unrelated to steel corrosion.

### 3.3. Configuration of R7 Sensor

As this experiment marked the inaugural use of the recently proposed POF sensor for corrosion detection, we focused on two key checkpoints to comprehensively understand the progression of corrosion within a steel plate. The initial checkpoint involved distributing observation areas across multiple locations spanning the entire steel plate to identify corrosion occurrences. Achieving this could be facilitated by installing the POF sensors a few centimeters apart, providing insights into the corrosion zone’s advancement across a broad expanse of the steel plate.

**Figure 13:**
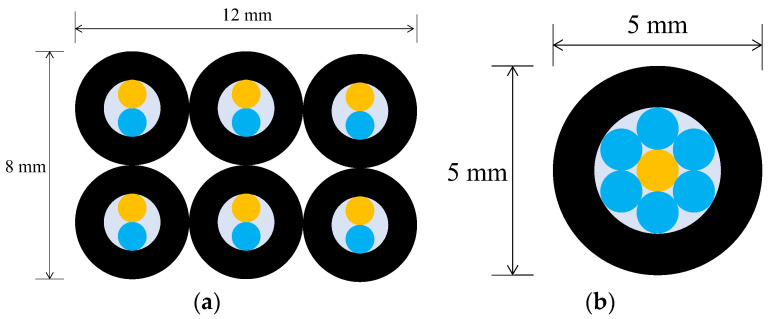
A comparison of two different strategies in the use of light emitting POF depicted by an orange color: (**a**) A strategy of having a light emitting POF in each R2 sensor; (**b**) A strategy for sharing a light emitting POF by multiple POFs.

The secondary checkpoint involved closely monitoring the progression of corrosion in the vicinity of each of the sensor installation points within the steel plate. To realize this, employing the previously described R2 sensor would entail utilizing two 1 mm-diameter POF cables within one sensor unit. If the pipes accommodating these POF cables had an inner diameter of 2 mm and an outer diameter of 4 mm, observing 6 locations would necessitate an 8 mm by 12 mm rectangular area (Figure 13a). In this case, the actual observation points would be spaced apart by a few millimeters. By implementing the concept of sharing a single POF illuminating multiple observation points, the arrangement depicted in Figure 13b enabled densely positioning six observation POFs within a circle approximately 5 mm in diameter (corresponding to the outer pipe diameter holding the POFs). This configuration facilitated the acquisition of detailed insights into corrosion progression at multiple contiguous locations in front of a single sensor unit.

**Figure 14:**
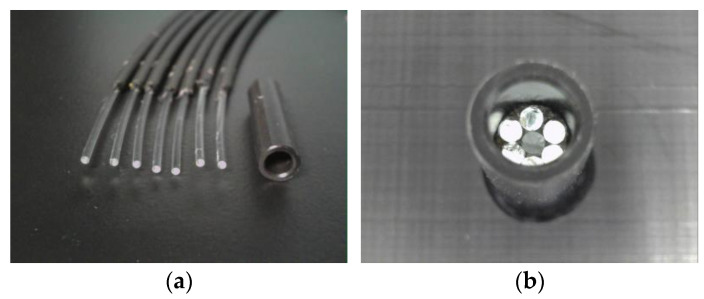
Structure of the R7 sensor: (**a**) Seven POF cables with a length of 2.5 m and a plastic pipe; (**b**) Assembled form before injecting adhesive A.

Figure 14 illustrates the preparation of seven POFs placed within a plastic pipe (inner diameter: 3 mm; outer diameter: 5 mm; length: 20 mm). Adhesive A (BD-SKCJ produced by BONDIC), which solidifies under ultraviolet (UV) irradiation, was introduced into the pipe up to a distance of 3 mm from the POF tips. The sensor is denoted as R7, signifying its construction with seven POFs.

The 3 mm space from the tip of the seven POFs within the holder pipe, having an inner diameter of 3 mm, required protection from water intrusion during the measurement period. To achieve this, adhesive A, capable of rapid solidification through UV irradiation, was employed to fill this space. Upon application of the adhesive into the designated area, any potential presence of air bubbles within the adhesive might adversely affect light-based measurements. Hence, if bubbles were found, they were cautiously removed using an ultrafine needle before UV light exposure for solidification. Subsequently, after assembling the sensor body, swift fixation onto the hole on the steel plate surface was essential; hence, adhesive B (Product ID #04612 produced by KONISHI BOND), known for instantaneous solidification, was utilized.

**Figure 15:**
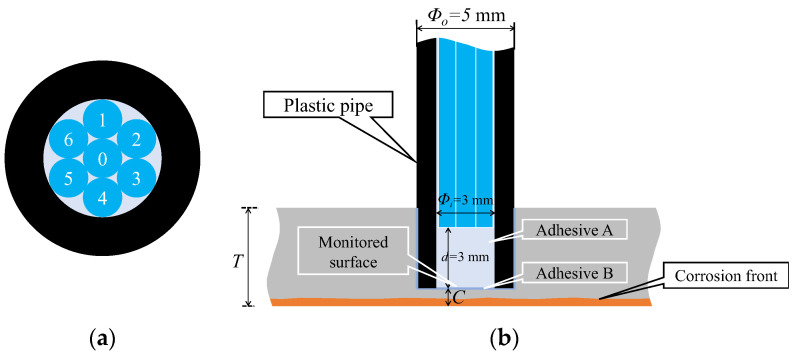
Structure and installation image of the R7 sensor: (**a**) Cross section; (**b**) Vertical cross section of the R7 sensor.

Figure 15 shows a detailed perspective of the R7 sensor. The seven POFs were tightly fitted within a plastic pipe with a 3 mm inner diameter, facilitated by the 1 mm diameter of each POF. The centrally positioned Fiber 0 was the light emitter, whereas the surrounding six fibers (Fibers 1–6) captured the light that was reflected and scattered from marginally varied points ahead of the sensor. Using a small quantity of general-purpose instant adhesive B, the R7 sensor was affixed into a 5 mm-diameter hole on the upper surface of a steel plate with thickness T. The R7 sensor facilitated the monitoring of a 3 mm diameter observation area (corresponding to a corrosion depth labeled as C, measured from the underside) to track the state and progression of the upward-propagating corrosion area initiated from the underside of the steel plate.

### 3.4. Locations of Light Irradiation and Reflection Reading

**Figure 16:**
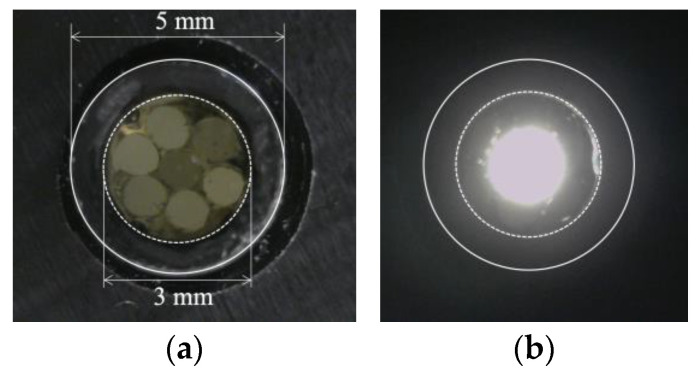
R7 sensor before and after transmitting light through Fiber 0: (**a**) R7 sensor before sending light into Fiber 0; (**b**) Zone illuminated by Fiber 0.

Figure 16 shows a frontal close-up of the R7 sensor (Figure 16a) and the state of the R7 sensor when the light was applied only to Fiber 0 (Figure 16b). 

**Figure 17:**
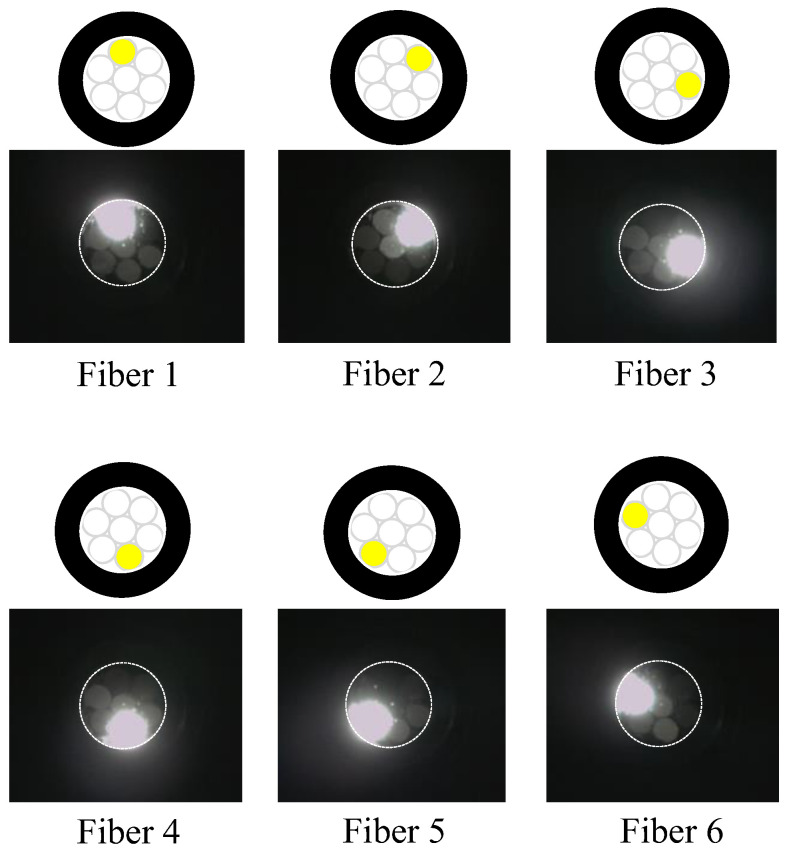
Zones illuminated by Fibers 1 to 6.

Evidently, the light emitted from Fiber 0 evenly illuminated the entire peripheral surface (with a diameter of 3 mm) of adhesive A, with a particular emphasis on a region approximately 2 mm in diameter (referred to as “Area 0”). Figure 17 shows the scenario where each of the surrounding POFs was illuminated, from Fibers 1 to 6. For example, the region designated as “Area 1”, illuminated by the light from Fiber 1, was not perfectly circular. This non-uniformity might be attributed to some of the light emitted by Fiber 1 being reflected by the inner surface of the plastic pipe before reaching the end of adhesive A.

**Figure 18:**
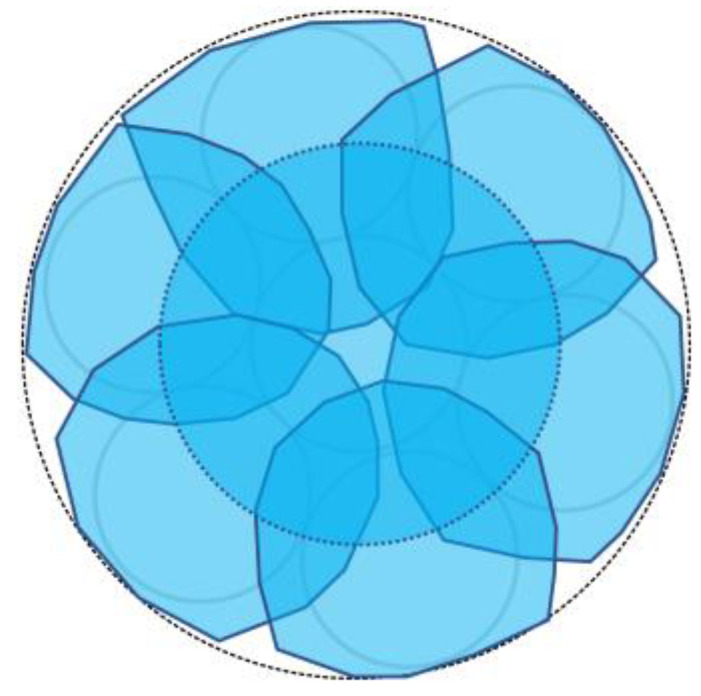
Observation zones for Fibers 1 to 6.

We considered a reverse approach in this context. When the light from Fiber 0 reached Area 0 on the adhesive surface A, some light was reflected or scattered. Among this reflected or scattered light, only the portion directed toward Fiber 1 and overlapping with Area 1 enters Fiber 1. In essence, Area 1 served as both the region where light entered Fiber 1 when it was irradiated and the region from which light in the surrounding areas entered Fiber 1 after being scattered or reflected. Thus, it became the “observed region perceived by Fiber 1”. Similarly, the respective observation areas of Fibers 2–6 could also be defined, as illustrated in Figure 18. This figure illustrates that the light emitted from Fiber 0 at the center could be employed to monitor the entire three-millimeter-diameter observation surface, with some overlap between the observation areas of Fibers 1–6.

### 3.5. Simulated Experiment Using R7 Sensor

**Figure 19:**
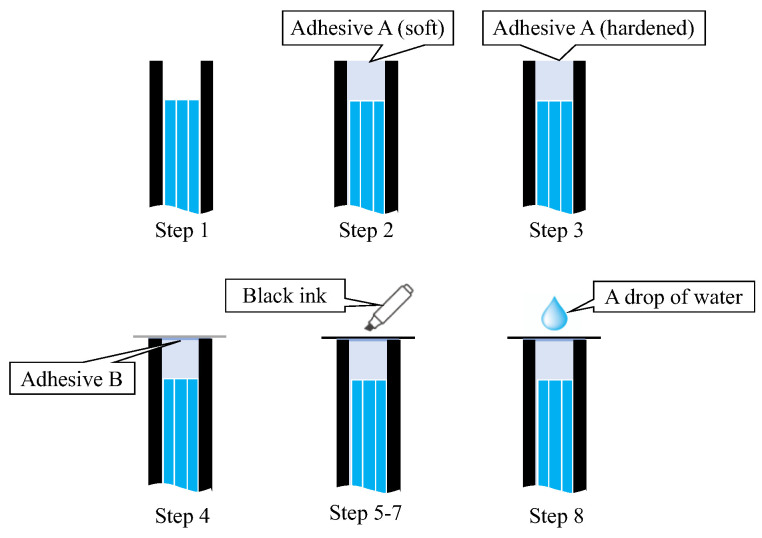
Sequence of actions performed to check the fundamental performance of the R7 sensor.

A simple experiment was conducted in a dark room to verify the functionality of the proposed R7 sensor before conducting the actual steel plate corrosion experiment. This experiment involved nine sequential steps (Steps 0–8) to assess the performance of the R7 sensor at each stage, as shown in Figure 19.

Step 0: Initially, the R7 sensor was incomplete, with only seven POFs inserted into the plastic pipe. The measurement commenced with no obstruction in front of the sensor, and this condition was maintained for approximately 20 s.
Step 1: Light was transmitted through Fiber 0 and emitted into the air.
Step 2: Adhesive A was applied to the 3 mm tip of the R7 sensor, and the sensor was maintained without exposure to UV light for 20 s.
Step 3: Then, adhesive A was exposed to UV light for solidification. During this process, the UV light directly affected the POF and disturbed the light intensity, rendering it unsuitable for graphical representation. The sensor was held in place for approximately 20 s following this process.
Step 4: A circular piece of paper with a diameter of 3 mm was cut from regular copier paper, colored gray (to simulate the color of steel), and affixed to the R7 sensor using adhesive B (a commercially available instant adhesive). The paper was glued with the gray side facing downward.
Steps 5–7: These steps involved incrementally marking the top surface of the paper (white side) using an oil-based black marker pen. This procedure ensured that the entire surface of the paper was eventually covered with black ink, which subsequently penetrated the bottom surface and was detected by the R7 sensor.
Step 8: Finally, a droplet of water was applied to the paper to assess the impact of moisture.

**Figure 20:**
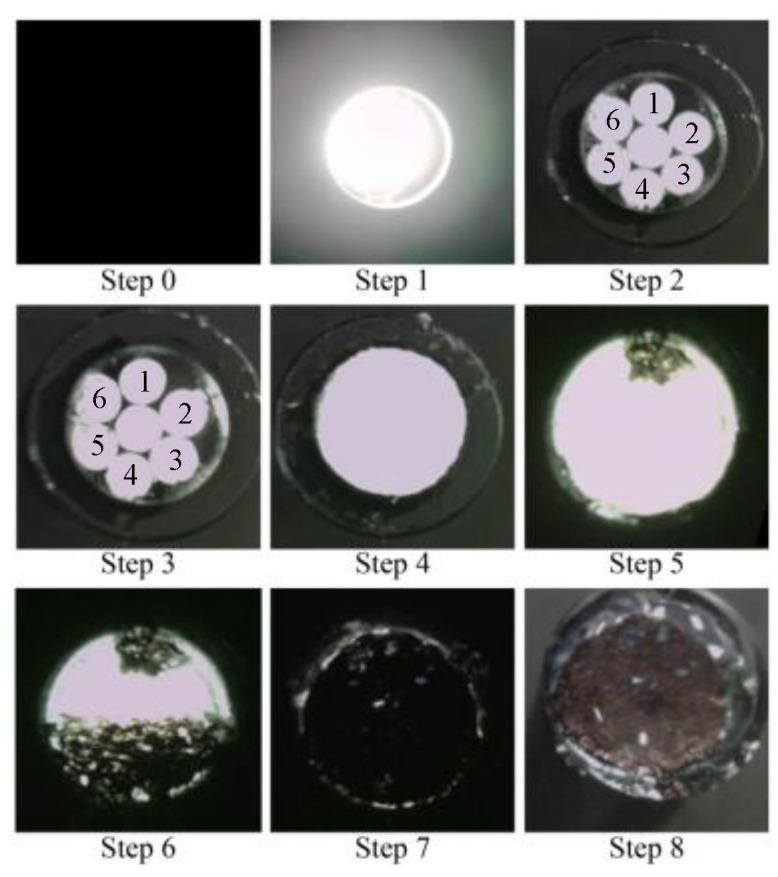
Photographic images of the fundamental experiment.

A detailed record of this process is illustrated in Figure 20. Note that the room lighting was switched on in certain instances, permitting a controlled amount of light to enter the POF within the sensor as needed. This was performed to facilitate the assessment of the sensor tip under specific conditions.

**Figure 21:**
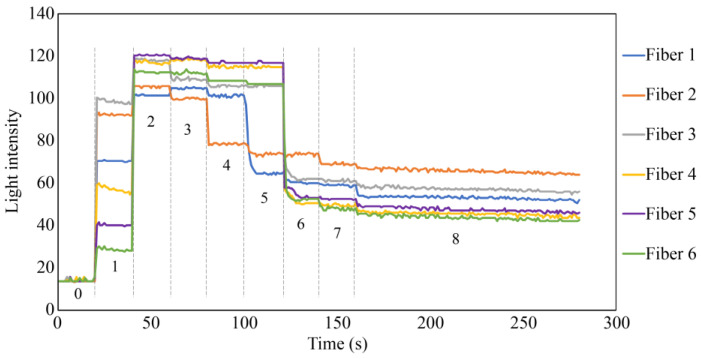
Light intensities recorded by Fibers 1 to 6 in the fundamental experiment.

Figure 21 shows the results of the simulated experiment. In Step 1, the diffused reflected light due to the micro-unevenness in the POF cross section and the plastic inner wall entered Fibers 1 to 6. The considerable variance in light intensity captured by Fiber 1 through Fiber 6 could be attributed to the imperfect flatness of the end faces of the seven POFs. The minor differences in the vertical positioning of the POFs’ end faces might also result in each POF surrounding Fiber 0 receiving diffused reflected light of slightly different intensities. In Step 2, the UV-curable adhesive (adhesive A) was applied to the tips of the seven POFs at a depth of approximately 3 mm. Consequently, light was reflected from their surfaces, significantly increasing the light intensity in Fibers 1–6. Subsequently, UV irradiation (the light intensity during irradiation was omitted from the graph because of the significant disturbance) solidified adhesive A, and a subtle change in the refractive index of the light was observed in Step 3.

In Step 4, a small quantity of commercially available instant adhesive B was applied to the upper surface of the solidified adhesive A. On top of this, a piece of paper with a gray underside was positioned. This procedure marginally reduced the light detected by Fibers 1–6 compared to that in Step 3. The initial state following the conclusion of Step 4 was analogous to the initial state in an actual corrosion experiment involving steel plates. The significant reduction in light intensity captured by Fiber 2 at this stage could have occurred due to the imperfectly flat edge surface of adhesive A and the boundary surface between adhesive B and the paper, attributed to the sensor’s handmade nature. Additionally, microscopic unevenness of the paper surface may potentially complicate light reflection and dispersion, resulting in varied light intensity levels received by Fibers 1 to 6, not necessarily equating in intensity.

In Step 5, a tiny dot was marked near the observation area of Fiber 1 using a black oil-based marker pen. In Step 6, approximately one-half of the area opposite the black dot corresponding to the space covered by Fibers 3–5 was filled in with the ink of the marker pen. In Step 7, the remaining areas were covered by the black ink, making the entire underside of the paper black. Throughout Steps 5–7, the light intensity in Fibers 1–6 decreased, corresponding to the blackened areas. Finally, to assess the impact of moisture, a water droplet was applied to the paper, allowing it to permeate through the paper. The light intensity in Fibers 1–6 was marginally reduced owing to this effect, confirming that the light intensity exhibited minor fluctuations owing to moisture, even when the color in front of the sensor remained constant. 

As demonstrated in this simulation, the proposed R7 sensor could precisely identify alterations in the condition of the object surface in front of it, primarily focusing on variations in the reflected light intensity; moreover, it could also sense changes in moisture levels.

## 4. Corrosion Experiment Utilizing a Steel Plate

### 4.1. Specifications of Steel Plates Utilized in the Experiment

**Figure 22:**
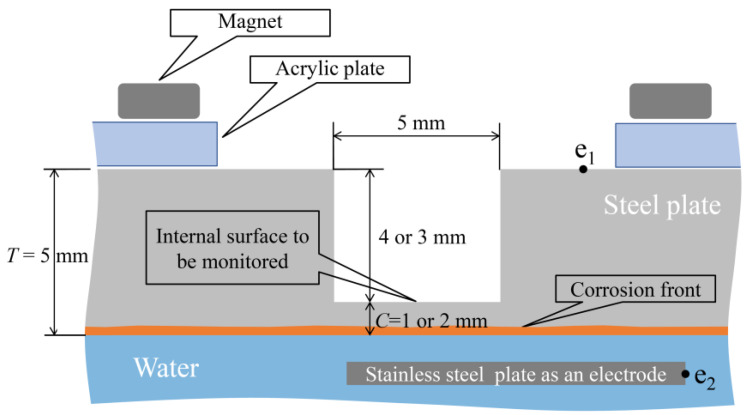
Fundamental strategy for the corrosion test.

In the corrosion experiment involving an aqueous solution system, a 5 mm-thick SS400 steel plate whose lower surface was submerged in water was utilized, as shown in Figure 22. The corrosion initiation on the lower side of the plate could be observed using this setup; thus, the upward propagation of corrosion could be tracked till a designated corrosion thickness (1 or 2 mm) was attained. Multiple observation holes with diameters of 5 mm and depths of 3 or 4 mm were incorporated into the upper surface of the steel plate to facilitate this. Some holes were equipped with R7 sensors, whereas others were left without a sensor to enable direct visual observation from above. Under these conditions, points e_1_ and e_2_, as shown in the figure, were linked to a DC electric circuit to conduct the electric corrosion test. The setup involved the preparation of an acrylic jig, onto which a neodymium magnet was affixed. This magnet secured the steel plate to a predetermined height.

**Figure 23:**
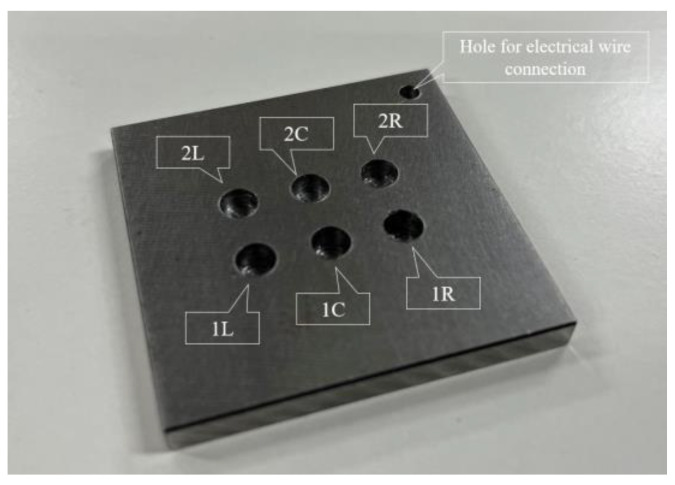
Steel plate used for the corrosion test with 6 holes for either POF sensor installation or direct visual observation. The depths of holes 1L, 1C, 1R are 4 mm; therefore, the remaining thickness of steel to be corroded is 1 mm. The depths of holes 2L, 2C, 2R are 3 mm; therefore, the remaining thickness of steel to be corroded is 2 mm. To make these holes, a drill bit with flat end was used.

**Figure 24:**
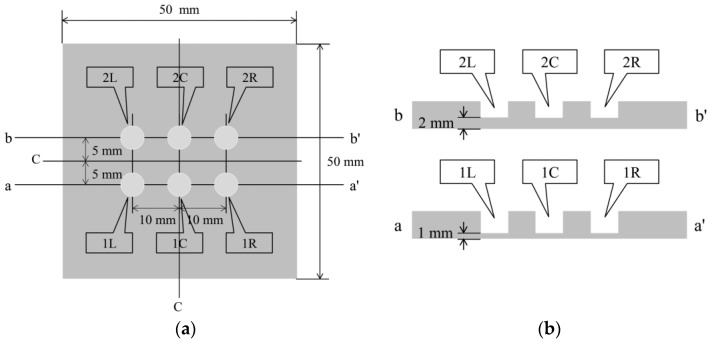
Details of the plate used for the corrosion test: (**a**) Top view of the six holes for R7 sensor installation and visual observation; (**b**) Cross-sectional views of the plate.

Figure 23 shows a steel plate measuring 50 mm × 50 mm × 5 mm composed of observation holes with unique notations. In each notation, the number denoted the thickness (in mm) of the steel plate remaining below the holes, while the letter signifies the location, where “L”, “C”, and “R” represented the left, center, and right sides, respectively. The thicknesses of the remaining steel plates, where the R7 sensor was installed, were deliberately set at 1 mm and 2 mm, respectively. This setup aimed to enable accurate detection of the corrosion depth precisely as it reached these individual thicknesses with a certain corrosion rate determined by a given set of experimental environments. The R7 sensors were positioned at 1R, 1L, 2R, and 2L among the six observation holes. In contrast, 1C and 2C were left open because these holes were designated for visual observation and image recording. The positions and cross-sectional views of the observation holes are shown in Figure 24.

### 4.2. Experimental Apparatus

**Figure 25:**
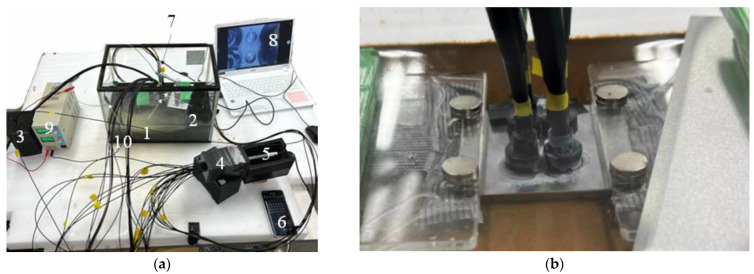
Overall layout of the plate corrosion test and a closer view of the plate: (**a**) Experimental configuration for the plate corrosion test: (1) Steel plate to be corroded. (2) R7 sensors. (3) Box containing an LED light source. (4) Box containing POF cables from the R7 sensors. (5) USB camera to capture images of the POF cables. (6) Mobile phone for graphic image analysis. (7) USB camera to observe the open holes. (8) PC for confirming and recording images of the open holes. (9) Direct current control unit. (10) Stainless steel plate; (**b**) Closer view of the sensor installation on the plate.

Figure 25 shows an overview of the complete experimental setup, featuring the steel plate with the R7 sensor installation. A 600 mm-wide, 300 mm-deep, and 400 mm-high glass container was filled with a 5% saline solution. Approximately half of the 5 mm-thick steel plate (1 in Figure 25a) was submerged in the saline solution. The R7 sensors were affixed to the top surface of the steel plate over observation holes 1R, 1L, 2R, and 2L using adhesive B. Light was transmitted from a white LED light source (3) to Fiber 0, which was positioned at the center of each R7 sensor. The light captured by the remaining six fibers was directed into a light-tight enclosure (4) that was designed to block external light and record internal light. Simultaneously, a USB camera (5) captured the images, which were then transmitted to a smartphone (6) equipped with the image processing application.

Another USB camera (7) was positioned above the observation holes 1C and 2C, which were left uncovered for visual observation to monitor the bottom of each hole. The resultant images were recorded on a PC (8). Moreover, a device designed to deliver DC for the electrolytic corrosion test (9) was configured. An electrical circuit was established by connecting the electrodes from this device to the steel plate, and the stainless steel plate (10) was immersed in the saline solution.

### 4.3. Experimental Results (Visual Observation)

**Figure 26:**
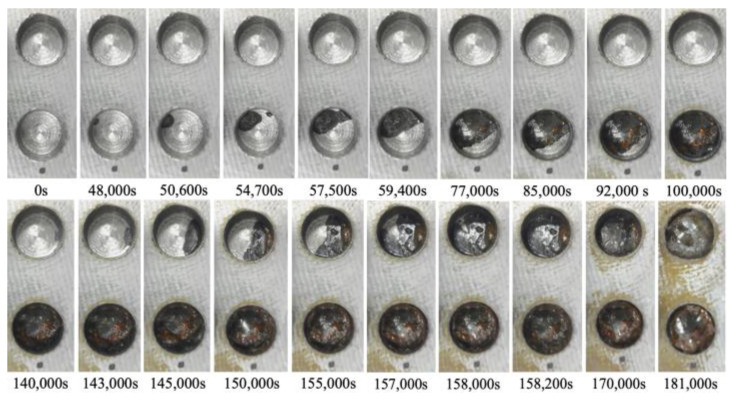
Images recorded at the open holes showing the progress of corrosion.

After the initial preparation, the electrolytic corrosion test was conducted at 30 V. It was concluded after approximately 50 h and 39 min (182,320 s) when satisfactory corrosion was confirmed in observation hole 2C, with a corrosion depth of 2 mm. Figure 26 shows the images captured at various stages of the corrosion experiment in observation holes 1C and 2C, which were designated for visual observation. The lower and upper photographs show 1C (thickness: 1 mm) and 2C (thickness: 2 mm), respectively. Approximately 48,000 s into the experiment, a black area appeared on the upper left shoulder of 1C, signifying the onset of a part of the corrosion front originating from the underside of the steel plate at a corrosion thickness of 1 mm. The corrosion area expanded and connected with a black area that surfaced on the upper right side. At 59,400 s, approximately half of the bottom of the hole was corroded. At 77,000 s, a brown area emerged within the corroded region and grew alongside the black area. At 100,000 s, the entire bottom of the borehole was corroded. Borehole C1 exhibited continuous development of black and brown corrosion products, reflecting the actual progression of the aqueous solution-based corrosion experiment.

Conversely, at 140,000 s, a corrosion zone emerged on the right side of 2C. The space occupied by the black and brown corrosion products increased, ultimately enveloping the entire area of 2C at 158,200 s. This indicated an ongoing escalation of corrosion products until the final stage.

Based on these visual observations, the times required to identify the initial corroded area in boreholes 1C and 2C with corrosion thicknesses of 1 and 2 mm were 48,000 and 140,000 s, respectively, indicating different corrosion thicknesses. Subsequently, the time taken for the corrosion zone to encompass the entire area, following its initial observation at the base of the borehole, was 52,000 and 18,200 s at 1C and 2C, respectively. These findings confirmed the fundamental corrosion initiation and propagation patterns observed in this experiment. However, the corrosion rate might not be uniformly distributed across the entire steel plate.

At approximately 77,000 s, the surface of the steel plate, which was the primary focus of this study, began to exhibit a light brown coloration, which progressively increased. This occurrence suggests that atmospheric corrosion coincided with the advancement of electrolytic corrosion in the aqueous solution system.

### 4.4. Experimental Results (R7 Sensor)

Visual inspection of observation holes 1C and 2C verified that the corrosion phenomena originating from the underside of the steel plate and advancing upward generally followed a similar sequence. However, this rate was not constant across different locations. Therefore, the proper functioning of the installed R7 sensors must be ascertained to monitor the progression of corrosion.

**Figure 27:**
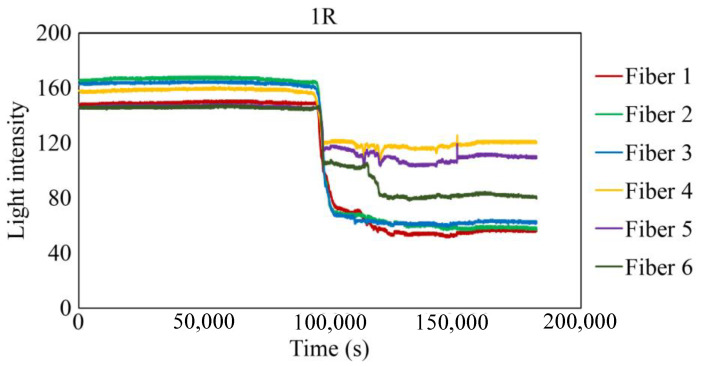
Light intensities recorded for the R7 sensor installed in 1R.

The behavior recorded by the R7 sensor in observation hole 1R is shown in Figure 27. Immediately after the commencement of the experiment, a consistent level of light was captured by Fibers 1–6. The light emitted from Fiber 0 penetrated the two adhesive layers and was reflected from the steel surface at the base of the observation hole 1R, providing stable measurements. Subsequently, within a specific timeframe preceding 100,000 s, the light intensities in Fibers 1–6 exhibited diminishing trends, albeit to varying degrees, and then remained somewhat unstable until approximately 150,000 s. Throughout this interval, the light intensities of Fibers 1 and 6 were unstable, particularly that of Fiber 6. This period is presumably influenced by corrosion byproducts, indicated by a blend of black, brown, and other hues, and possibly water, generated by the ongoing corrosion process. Moreover, the light intensities generally stabilized beyond 150,000 s, signifying that the conditions in front of Fibers 1–6 became steady and independent of the progression of corrosion.

**Figure 28:**
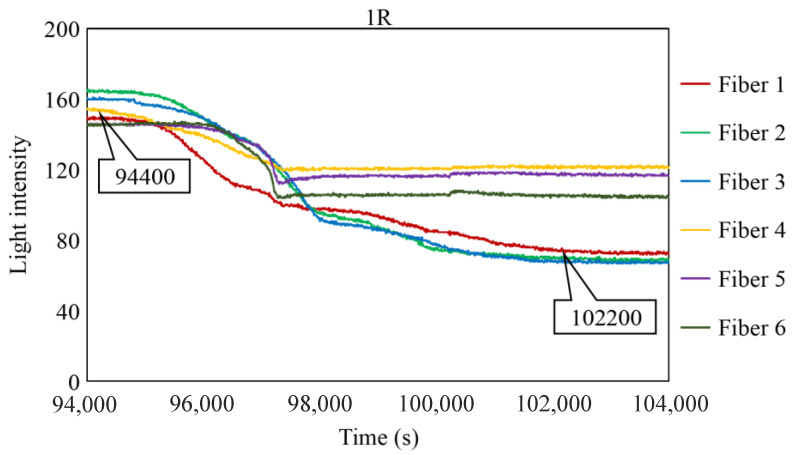
Light intensities recorded by the R7 sensor installed in 1R from 94,000 to 100,000 s.

To scrutinize the precise timeframe when the corrosion front reached the base of borehole 1R, the interval from 94,000 to 104,000 s was meticulously examined, and the results are shown in Figure 28. The figure illustrates scenarios in which the reduction in light intensity occurred at varying paces; for example, Fibers 1 and 4 experienced a relatively gradual decrease, whereas Fibers 5 and 6 exhibited a swift decline. Among the six fibers, Fiber 4 exhibited the earliest decrease in light intensity with an estimated elapsed time of approximately 94,400 s, denoted as C_i_, signifying the earliest corrosion area coverage in this observation hole. The decline in light intensity progressed within each observation fiber, and the point at which the diminishing trend was presumed to have mostly halted was labeled as C_f_, occurring at approximately 102,200 s in Fiber 1. Consequently, the concurrent reduction in light intensity was construed as a progression wherein the corrosion front initially reached a specific point on the borehole bottom in 1R and then subsequently spread across the entire borehole bottom within a timeframe of ΔT_c_ (= C_f_ − C_i_), which was approximately 7800 s in the case of 1R. Notably, this duration was significantly shorter than the corresponding periods in boreholes 1C (ΔT_c_ = 52,000 s) and 2C (ΔT_c_ = 18,200 s), suggesting the formation of a relatively uniform corrosion front within the circular area of 3 mm diameter that was monitored by the R7 sensor installed in 1R.

The light intensity at the end of the experiment fluctuated within a range of 60–120. Provided that a part of the observed light, described as *L_c_* in Figure 12, remained unaltered throughout the experiment, we can deduce that corrosion byproducts exhibiting subtle variations in hues were consistently present within the observation regions of Fibers 1–6 as the corrosion process neared completion.

**Figure 29:**
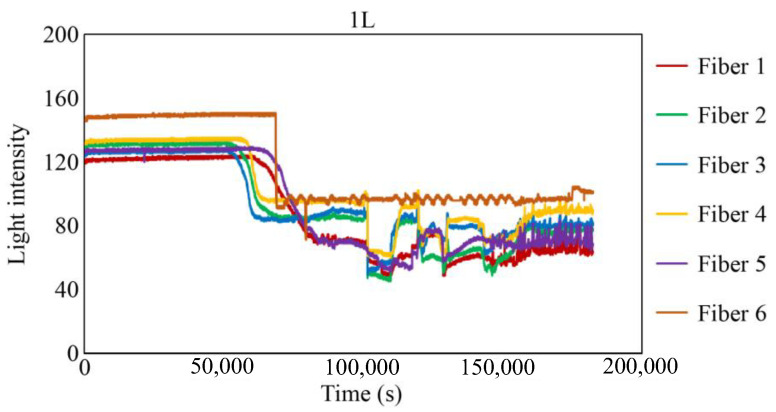
Light intensities recorded by the R7 sensor installed in 1L.

**Figure 30:**
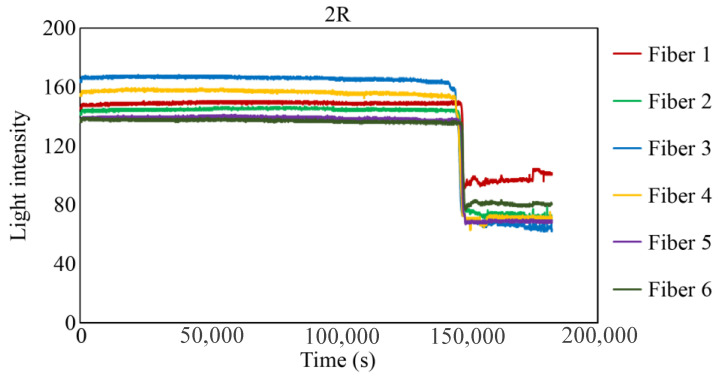
Light intensities recorded by the R7 sensor installed in 2R.

**Figure 31:**
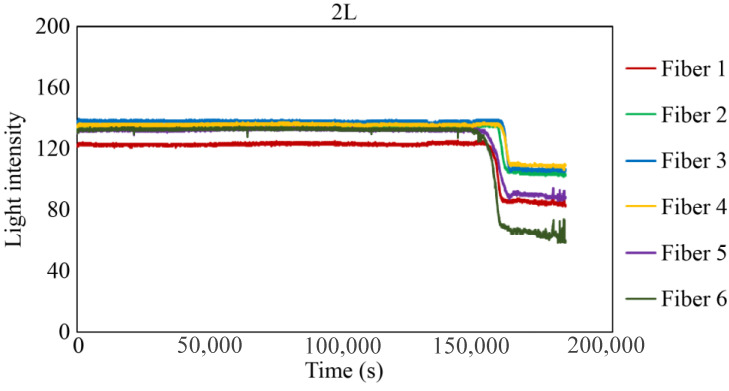
Light intensities recorded by the R7 sensor installed in 2L.

**Figure 32:**
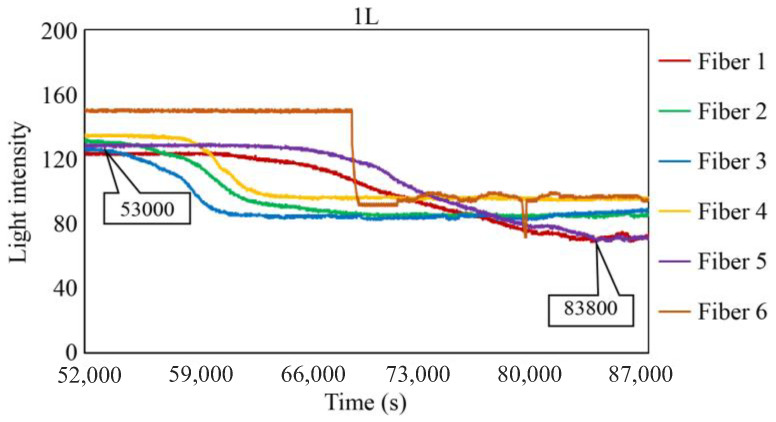
Light intensities recorded by the R7 sensor installed in 1L from 50,000 to 78,000 s.

**Figure 33:**
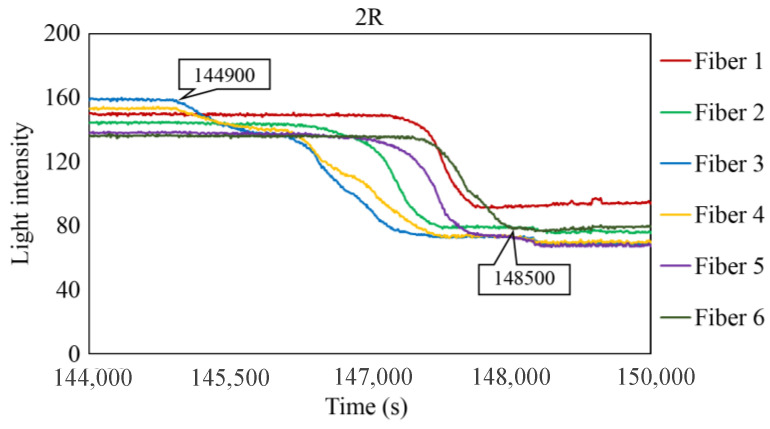
Light intensities recorded by the R7 sensor installed in 2R from 144,000 to 149,000 s.

**Figure 34:**
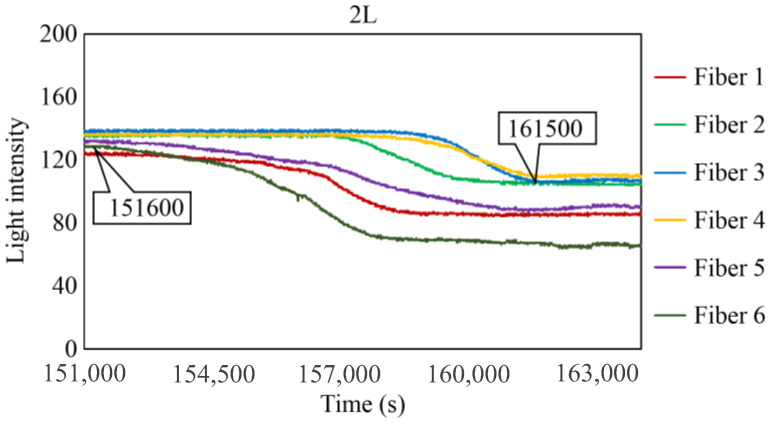
Light intensities recorded by the R7 sensor installed in 2L from 152,000 to 164,000 s.

Figure 29, Figure 30 and Figure 31 show the light intensity results recorded in additional observation holes, namely, 1L, 2R, and 2L. In 1L, the light intensity in certain fibers decreased considerably earlier than in 1R despite having the same corrosion thickness. Subsequently, the light intensity exhibited irregular fluctuations during the simultaneous decrease and in the subsequent period, where it fluctuated but remained stable. This suggests that the generation of corrosion products near observation hole 1L was highly intricate. The irregular light intensity fluctuations persisted until just before the experiment concluded. In boreholes 2R and 2L, where the corrosion thickness measured 2 mm, a simultaneous decrease in light intensity occurred around 150,000 s, followed by stabilization. Figure 32, Figure 33 and Figure 34 provide magnified images of the period during which the simultaneous light intensity reduction occurred in 1L, 2R, and 2L. These boreholes exhibited corrosion areas appearing at marginally different times in various locations, indicating the recording of the corrosion development process.

Table 1 summarizes the time C_i_ when corrosion was initially observed in each borehole, the time C_f_ when the simultaneous light intensity reduction was finalized, covering the entire borehole with corrosion, and ΔT_c,_ which is the time difference between the two, denoted as C_f_ − C_i_.

The average values of C_i_ in boreholes 1R and 1L with a 1 mm corrosion thickness and in boreholes 2R and 2L with a 2 mm corrosion thickness were calculated to be 73,700 and 148,250 s, respectively. The ratio of the two is approximately 1:2, indicating that the difference in the corrosion thickness is suitably mirrored in the variations in the detection time of the occurrence of corrosion. Furthermore, the actual arrival time of the corrosion zone ranged from 3600 to 78,000 s, even with the same corrosion thickness.

### 4.5. Raw Image Captured by the Image Processing Application of the R7 Sensor

**Figure 35:**
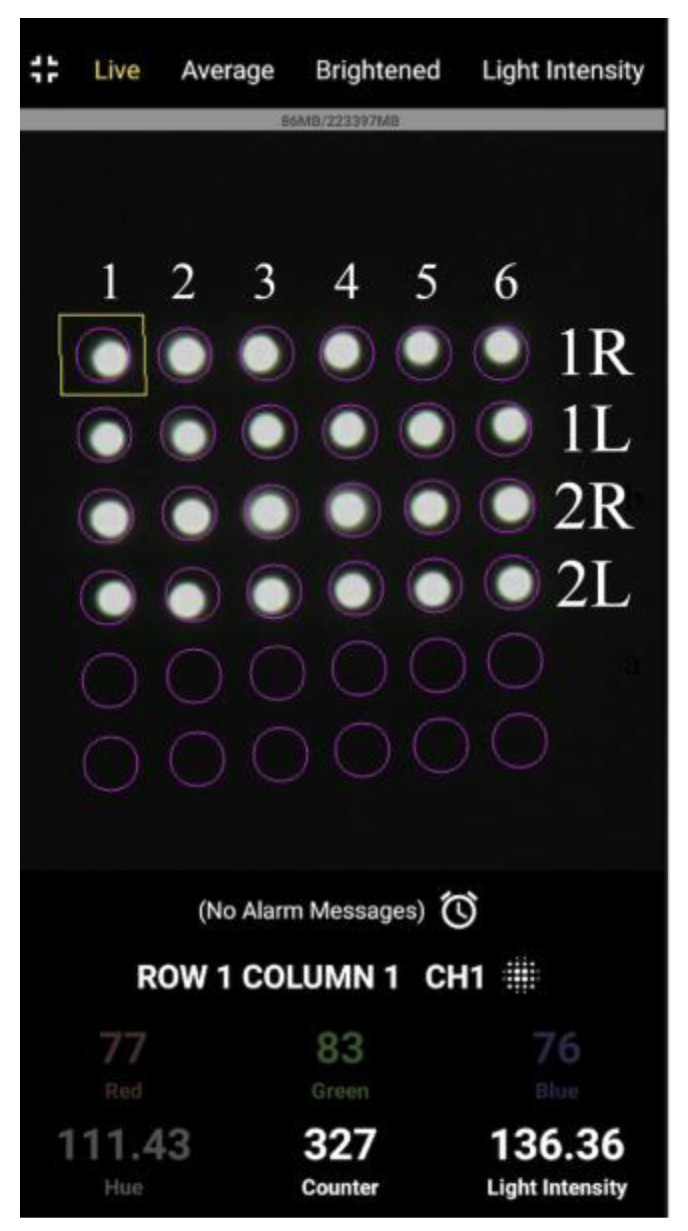
Screenshot image of the light observed for R7 sensors at the beginning of the test.

The focus of the analysis up to this point had been the light intensity captured by the R7 sensor. However, this section described the image processing application utilized, which could capture screenshots (Figure 35) showing the conditions of the POFs during monitoring. In this screenshot, the top row shows six lights representing Fibers 1 (leftmost) to 6 (rightmost) of the R7 sensor installed at observation hole 1R. In contrast, the second, third, and fourth rows correspond to observation holes 1L, 2R, and 2L, respectively.

**Figure 36:**
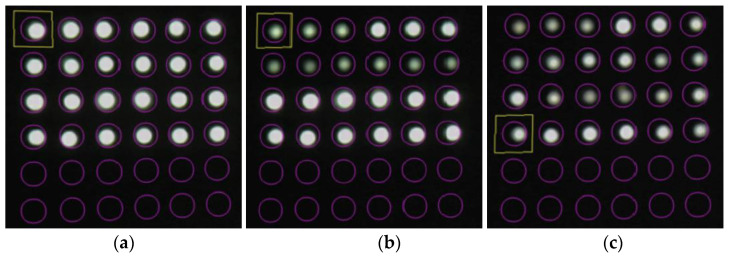
Screenshot images of the light observed for R7 sensors during the test: (**a**) 0 s; (**b**) 107,000 s; (**c**) 181,000 s.

Figure 36 shows screenshot images recorded at the start of the experiment (elapsed time: 0 s), when the corrosion thickness was believed to have surpassed 1 mm (elapsed time: 107,000 s), and after the experiment (elapsed time: 181,000 s).

Compared with the initial light brightness recorded, the light in observation holes 1R and 1L became dimmer at the 107,000-s mark, signifying that the simultaneous decline in light intensity had predominantly ceased in these holes. In the final stage of the experiment, at 181,000 s, a concurrent drop in light intensity occurred in boreholes 2R and 2L, despite certain POFs exhibiting relatively higher brightness. Notably, if a detailed analysis was unnecessary for a given project, a basic recognition of the corrosion phenomenon could be achieved solely through visual inspection. 

## 5. Possibilities and Challenges of Corrosion Monitoring Using POF

**Figure 37:**
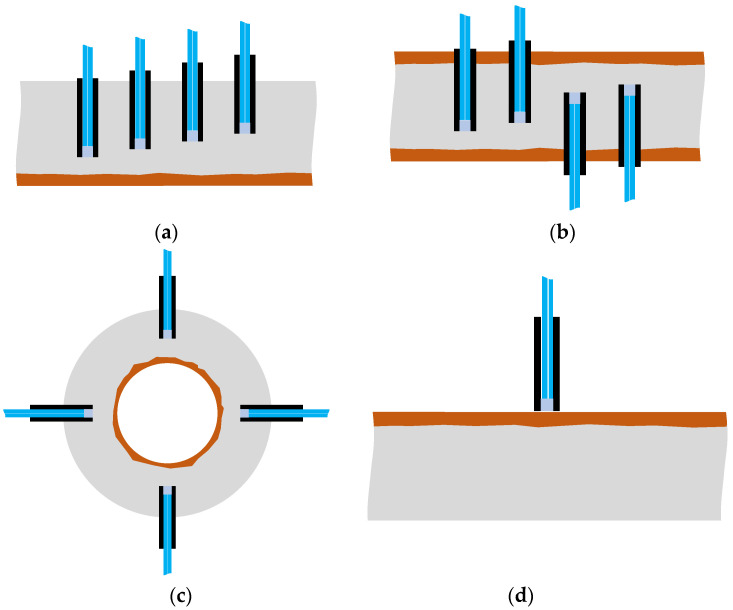
Various configurations for corrosion monitoring using POF sensors: (**a**) R2 sensors for monitoring the progress of corrosion at multiple depths; (**b**) R2 sensors for monitoring the progress of corrosion from two directions; (**c**) R2 sensors for monitoring the progress of corrosion in pipes; (**d**) R2 sensor to be used for corrosion detection on the surface of a plate.

In this study, we focused on the phenomenon in which light reflected on the surface of an object could be captured to obtain insight into the changes in the surface state of an object. A POF sensor was used to identify the expansion of the corrosion area originating from one side of a steel plate and progressing in the opposite direction. The experiment utilized a specific configuration (R7 sensor) in which the sensor was composed of one light-supplying POF and six POFs for light observation, thereby allowing detailed monitoring of the evolving corrosion zone. However, in practical applications, a sensor with seven POFs need not be installed at each corrosion-checking location; the simpler R2 sensor equipped with two POF cables will be sufficient. For a more detailed determination of the corrosion zone depth, R2 sensors could be installed at various locations, as shown in Figure 37a. Alternatively, if corrosion is expected to occur and propagate from both sides of a steel plate, R2 sensors could be positioned on both sides, as illustrated in Figure 37b. The arrangement shown in Figure 37c could be used to monitor a corrosion zone expanding outward from the interior of a thick-walled circular pipe, provided the sensor installation did not disturb structural safety. This paper introduces a novel POF sensor designed initially for monitoring corrosion progression on a metal plate; however, it is also applicable for detecting surface corrosion. For this application, the adhesive would be selectively applied solely to the sensor’s front surface to block water penetration, consequently averting corrosion. Thus, as depicted in Figure 37d, the sensor should be used adhesive-free, ensuring a slight gap existed between the sensor’s front surface and the targeted monitoring point.

**Figure 38:**
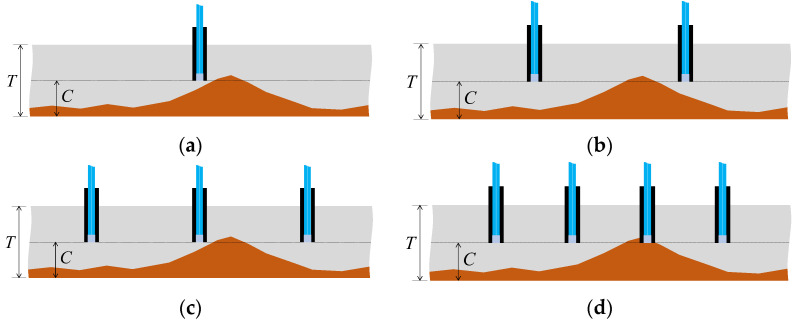
Monitoring strategy comparison with uneven progress of the corroded region: (**a**) Monitoring with 1 sensor; (**b**) Monitoring with 2 sensors; (**c**) Monitoring with 3 sensors; (**d**) Monitoring with 4 sensors.

Considering scenarios where corrosion thickness or rates vary across locations and the goal was to detect the point when the corrosion depth reached *C* at the most advanced area of the plate (thickness *T*), deploying only one sensor might lead to a high probability of failure to detect the corrosion front, as depicted in Figure 38a. Even with an increase in the number of sensors to 2 or 3 (Figure 38b,c), there remained a possibility of missing the corrosion front. Increasing the number of sensors, regardless of cost or time, naturally enhanced the chance of one sensor detecting the corrosion zone front (Figure 38d). However, solely increasing the number of sensors to elevate the probability of detecting the corrosion zone or identifying corrosion rates raised multifaceted concerns. These included considerations of budgetary constraints for monitoring and structural safety implications due to the puncturing procedures required for installing the sensors on the target structure. To address these challenges, strategies like minimizing sensor diameter to reduce costs and mitigating structural safety impacts during installation were under contemplation for future investigation.

Furthermore, based on the principle of light reflection, the POF sensor proposed in this study can be produced at a low cost and does not require explosion-proof measures. It also exhibits superior long-term durability, as both the sensor and necessary fixtures can be crafted from plastic materials. These sensors must be extensively deployed at critical monitoring points on various structures that require corrosion monitoring to efficiently and comprehensively assess the health of these structures through the captured light. The image processing application introduced in this paper can process multiple POF sensors (a single smartphone can handle up to 900 sensors), enabling the acquisition of digital data on the onset and progression of corrosion from numerous locations. Automated processing of monitored data would also become possible through an integrated utilization of ICT technologies together with the proposed image processing application. Moreover, the results indicate that this light-based method is adequate for visual inspection, particularly when only a rough estimation of the corrosion phase is required. Therefore, if R2 sensors are positioned at numerous pivotal monitoring points, an inspector can check the light captured by the R2 sensor on-site whenever necessary using a portable light source (e.g., an LED flashlight). This offers an efficient and cost-effective means of verifying the structural corrosion state by comparing it to healthy conditions. While the laboratory experiments provided detailed data analysis using multiple POF cables, real-world applications should prioritize cost efficiency and simplicity. Moreover, deploying POF sensors at multiple points on actual structures may raise concerns regarding the POF length, in addition to the persistent challenges associated with the relatively higher light attenuation in plastic optical fibers than in glass optical fibers. Addressing these issues requires further research and development to exploit the full potential of POF sensors for efficiently monitoring a wide range of infrastructure assets in the field.

## 6. Conclusions

In this study, we addressed the challenge of comprehending the corrosion conditions of steel, which was a crucial concern for engineers in an era where substantial infrastructure must be efficiently maintained. We developed a cost-effective sensor composed of POFs that could be applied at multiple points and verified its functionality through laboratory experiments. The conclusions drawn from this study were as follows:

1. The fundamental capability of the R2 sensor, which was composed of two POFs, was validated. The experimental results determined that a distance of approximately 3 mm between the tip of the sensor and the surface of the object was adequate for accurately detecting changes occurring on the surface of the object.
2. When using the R7 sensor, which comprises seven POFs, the alterations to the surface condition could be effectively monitored through two transparent adhesive layers positioned between the sensor and the object’s surface. However, careful analysis of the optical data was essential because the measured light contained reflections and transmissions that occurred through the two adhesive layers, precluding direct observation of the object’s surface.
3. During the corrosion experiment conducted in an aqueous solution, where the onset of corrosion occurred at the underside of a five-millimeter-thick steel plate and progressed upward, the corrosion front did not exhibit uniform flatness but displayed marginal variations at different locations. This phenomenon was also observed through the visual observation holes on the surface of the steel plate and in the four observation holes equipped with the R7 sensor.
4. In the corrosion experiments conducted on steel plates, the R7 sensors placed at sites with corrosion thicknesses of 1 or 2 mm could detect the initial corrosion after a certain amount of elapsed time, which exhibited a proportional relationship with the thickness of the corrosion. The results indicated that approximately 3600–30,800 s elapsed from when the corrosion zone initially reached the bottom of the borehole until it was fully covered.
5. While the R7 sensor could observe a 3 mm-diameter circle within the borehole bottom (with a diameter of 5 mm), it could also identify subtle variations in the corrosion state based on the location. This discrimination was facilitated using Fibers 1–6, which were affixed to the R7 sensor. The R7 sensor could document dissimilarities in optical data arising from variations in the condition of the object and the color of the corrosion products, among other factors.
6. While assessing the corrosion state, the light collected by the POF sensor could be precisely documented using an image processing application installed on a mobile phone. We also established that, for a preliminary assessment, visual confirmation alone was sufficient to substantiate the presence of corrosion, which promised a cost-effective inspection procedure would be available for the global community of engineers working in the infrastructure maintenance business.

## Figures and Tables

**Table 1:** Timeline of the corrosion test.

Hole	Elapsed Time at Which Corrosion Was Detected for the First Time: C_i_	Elapsed Time at Which the Reduction in Light Intensities Was Stabilized: C_f_	Time Required for the Reduction in Light Intensities: ΔT_c_ = C_f_ − C_i_
1R	94,400 s	102,200 s	7800 s
1L	53,000 s	83,800 s	30,800 s
2R	144,900 s	148,500 s	3600 s
2L	151,600 s	161,500 s	9900 s

## Data Availability

Data is contained within the article.
